# Sequential infection can decrease virulence in a fish–bacterium–fluke interaction: Implications for aquaculture disease management

**DOI:** 10.1111/eva.12850

**Published:** 2019-08-14

**Authors:** Anssi Karvonen, Andy Fenton, Lotta‐Riina Sundberg

**Affiliations:** ^1^ Department of Biological and Environmental Science University of Jyvaskyla Jyvaskyla Finland; ^2^ Institute of Integrative Biology University of Liverpool Liverpool UK; ^3^ Nanoscience Center University of Jyvaskyla Jyvaskyla Finland

**Keywords:** dynamic infection, epidemiology, multiple infections, sequential infection, spatiotemporal variation

## Abstract

Hosts are typically infected with multiple strains or genotypes of one or several parasite species. These infections can take place simultaneously, but also at different times, i.e. sequentially, when one of the parasites establishes first. Sequential parasite dynamics are common in nature, but also in intensive farming units such as aquaculture. However, knowledge of effects of previous exposures on virulence of current infections in intensive farming is very limited. This is critical as consecutive epidemics and infection history of a host could underlie failures in management practices and medical intervention of diseases. Here, we explored effects of timing of multiple infections on virulence in two common aquaculture parasites, the bacterium *Flavobacterium columnare* and the fluke *Diplostomum pseudospathaceum*. We exposed fish hosts first to flukes and then to bacteria in two separate experiments, altering timing between the infections from few hours to several weeks. We found that both short‐term and long‐term differences in timing of the two infections resulted in significant, genotype‐specific decrease in bacterial virulence. Second, we developed a mathematical model, parameterized from our experimental results, to predict the implications of sequential infections for epidemiological progression of the disease, and levels of fish population suppression, in an aquaculture setting. Predictions of the model showed that sequential exposure of hosts can decrease the population‐level impact of the bacterial epidemic, primarily through the increased recovery rate of sequentially infected hosts, thereby substantially protecting the population from the detrimental impact of infection. However, these effects depended on bacterial strain–fluke genotype combinations, suggesting the genetic composition of the parasite populations can greatly influence the degree of host suppression. Overall, these results suggest that host infection history can have significant consequences for the impact of infection at host population level, potentially shaping parasite epidemiology, disease dynamics and evolution of virulence in farming environments.

## 1 INTRODUCTION

Hosts are commonly infected with multiple parasite species or strains/genotypes of one species at the same time (Graham, 2008; Read & Taylor, 2001; Salgame, Yap, & Gause, 2013; Telfer et al., 2010). Such infections can result in direct (interference competition) or indirect (resource or host immune‐mediated competition) interactions between parasites and have significant implications for key parasite traits such as virulence, harm to the host (Bell, Roode, Sim, & Read, 2006; Ben‐Ami, Regoes, & Ebert, 2008; Davies, Fairbrother, & Webster, 2002; de Roode, Helinski, Anwar, & Read, 2005). Recent studies have emphasized the importance of multiple infections also between phylogenetically distant parasites (Ben‐Ami, Rigaud, & Ebert, 2011; Clay, Dhir, Rudolf, & Duffy, 2019; Doublet, Natsopoulou, Zschiesche, & Paxton, 2015; Duncan, Agnew, Noel, & Michalakis, 2015; Fellous & Koella, 2009; Lohr, Yin, & Wolinska, 2010; Vojvodic, Boomsma, Eilenberg, & Jensen, 2012), suggesting that interactions can take place at the scale of the entire parasite community of one host. It is common that infections from different parasites do not occur only simultaneously, but also sequentially at different times as the infection risk in nature varies over time (e.g. seasons [Faltýnková, Valtonen, & Karvonen, 2008; Karvonen, Seppälä, & Valtonen, 2004]) and space (e.g. spatial aggregation of infected hosts [Byers, Blakeslee, Linder, Cooper, & Maguire, 2008; Jokela & Lively, 1995; King, Delph, Jokela, & Lively, 2009]). The timing between different infections again can vary from a few hours to several weeks, or even years. Consequently, each individual host can have a different infection history and immunological status, thus making the landscape of disease outcomes complex and unpredictable. Empirical examples of sequential infections of multiple parasites in plants (Hood, 2003; Laine, 2011; Marchetto & Power, 2018), invertebrates (Ben‐Ami, Mouton, & Ebert, 2008; Ben‐Ami et al., 2011; Gower & Webster, 2005; Lohr et al., 2010; Natsopoulou, McMahon, Doublet, Bryden, & Paxton, 2015) and vertebrates (Graham, 2008; Hoverman, Hoye, & Johnson, 2013; Klemme, Louhi, & Karvonen, 2016) suggest an effect of sequential infection of hosts on parasite fitness‐related traits such as infection success and virulence.

Infections from multiple parasites are common also in intensive production environments, where high densities of susceptible hosts favour the spread of virulent pathogens (Kennedy et al., 2016; Pulkkinen et al., 2010). Infections can cause significant economic loss by impairing quality, condition and growth of crops and farmed animals. For example, in aquaculture, parasitic infections are considered one of the most important threats for development of the industry. Similar to natural conditions, parasitic epidemics in aquaculture are typically consecutive with different parasites infecting their hosts in varying timescales. Disease epidemics typically sweep through aquaculture units at different times in response to variation in pathogen ecology and host susceptibility (e.g. cohorts of varying age) (Karvonen, Rintamäki, Jokela, & Valtonen, 2010; Rintamäki‐Kinnunen & Valtonen, 1997). This creates favourable conditions for development of cumulative infection history of hosts that can affect virulence in subsequent disease outbreaks. Earlier studies in fish have shown that a prior parasite exposure can influence the outcome of simultaneous re‐exposure of the host by multiple parasite genotypes (Klemme et al., 2016), alter associations between parasite species (Karvonen, Seppälä, & Valtonen, 2009), and influence parasite community composition of the host (Benesh & Kalbe, 2016). However, knowledge of the implications of sequential exposure of hosts to multiple parasites in farming environments is still very limited. Consequently, most infections occurring in intensive farming units are commonly treated instantaneously with very little consideration of previous or existing other infections, which, among aquaculture fish, can range from viruses and bacteria (Mohanty & Sahoo, 2007; Pulkkinen et al., 2010; Skall, Olesen, & Mellergaard, 2005; Tobback, Decostere, Hermans, Haesebrouck, & Chiers, 2007) to protozoans and metazoans (Hakalahti & Valtonen, 2003; Karvonen, Savolainen, Seppälä, & Valtonen, 2006; Rintamäki‐Kinnunen & Valtonen, 1997). Regardless, infection history of a host population could influence ongoing epidemics and potentially underlie failures in management practices and medical intervention of diseases.

Here, we studied effects of host sequential exposure on parasite virulence in an interaction between two widely distributed aquaculture parasites, the bacterium *Flavobacterium columnare* and the fluke *Diplostomum pseudospathaceum*. Bacterium *F. columnare*, the causative agent of the columnaris disease, is an opportunistic pathogen and currently considered as one of the most severe disease threats in fish farming (Declercq, Haesebrouck, Van den Broeck, Bossier, & Decostere, 2013). The disease can cause considerable losses if not treated with antibiotics (Declercq, Haesebrouck, et al., 2013; Pulkkinen et al., 2010; Wagner, Wise, Khoo, & Terhune, 2002), which in many cases has resulted in emergence of antibiotic‐resistant bacterial strains (Declercq, Boyen, et al., 2013). The trematode *D. pseudospathaceum* causes local, but significant aquaculture problems by blinding fish (Karvonen, 2012). Unlike *F. columnare*, infections of *D. pseudospathaceum* are not transmitted directly between fish, but the life cycle includes three hosts (snail, fish and fish‐eating bird) and fish become infected when in contact with the parasite larvae (cercariae) released from infected snails. Infections of *F. columnare* and *D. pseudospathaceum* can co‐occur in aquaculture fish (Karvonen et al., 2006; Sundberg et al., 2016). They also interact in genotype‐specific manner when infecting the host at the same time, which can result in higher morbidity of fish, that is virulence, and higher infection success of the fluke (Louhi, Sundberg, Jokela, & Karvonen, 2015).

We first exposed rainbow trout (family Salmonidae) hosts to both parasites in two experiments manipulating the timing between the infections from few hours to several weeks and monitoring the disease‐related morbidity of the fish. Based on our previous results on simultaneous infections of the two parasites (Louhi et al., 2015), we expected that both the short‐term and the long‐term sequences between the infections would result in lower bacterial virulence, possibly depending on the strain–genotype combinations of the parasites. Similarly, we expected sequential infection to change infection success of the fluke. Second, we developed a compartmental mathematical model capturing the disease dynamics in a host population to explore how sequential exposure of hosts to the two parasites could influence disease‐related mortality, and total host population size, of farmed fish. Overall, our results suggest that host infection history can potentially shape parasite virulence over a long time period, which may have implications for evolution of virulence as well as for disease prevention strategies in intensive farming systems.

## 2 MATERIAL AND METHODS

### 2.1 Bacterial cultures

Three *F. columnare* strains (1–3, Appendix S1, Table S1) differing in their virulence were used (Kunttu, Sundberg, Pulkkinen, & Valtonen, 2012; Laanto, Bamford, Laakso, & Sundberg, 2012). The strains had originally been isolated from fish farms or from environment in 2008–2010 by standard culture methods using Shieh medium (Song, Fryer, & Rohovec, 1988) and Shieh medium supplemented with tobramycin (Decostere, Haesebrouck, & Devriese, 1997). Different sampling locations, sampling times, sources of isolation (fish vs. environment), ARISA groups (Table S1), differences in CRISPR‐Cas sequences (Laanto, Hoikkala, Ravantti, & Sundberg, 2017) and the different pathogenicity of the isolates (Kunttu et al., 2012; Laanto et al., 2012) ensured that the strains differed in genetic and/or ecological characteristics. Cultures were stored at −80°C with 10% glycerol and 10% foetal calf serum. Prior to the exposures, bacterial strains were grown overnight in 2 ml of Shieh medium, then enriched in 1:10 in fresh medium and incubated at 25°C with 150 rpm agitation for 22 hr. The optical density (OD, A570) of the culture was measured with spectrophotometer, and the corresponding colony‐forming units (CFU) were calculated using a previously determined relationship between OD and CFU (unpublished).

### 2.2 Sampling and genotyping of flukes

*Lymnaea stagnalis* snails (*n* = 42), intermediate hosts for *D. pseudospathaceum*, shedding clonal fluke cercariae were collected from Lake Vuojärvi (62°24′54″N, 25°56′14″E), Finland. Fifteen cercariae were collected from each snail and stored individually in Eppendorf tubes in 15 μl of lake water and frozen in −20°C for subsequent microsatellite analysis to identify snails that were infected with one fluke genotype (Louhi, Karvonen, Rellstab, & Jokela, 2010; Reusch, Rauch, & Kalbe, 2004) (Table S2). Parasite DNA was extracted according to Criscione and Blouin (2004). Snails infected with one genotype were stored individually in 1 L of water at 6°C and fed ad libitum with lettuce until the beginning of the experiment. Note that all parasite genotypes are produced sexually in the avian definitive host, which is why all infections in the snails are unique and individual genotypes persist in a host population only for one complete round of the parasite life cycle.

### 2.3 Experimental exposure 1

Naïve, uninfected juvenile rainbow trout (*Oncorhynchus mykiss*; age 2.5 months, average length ± *SE* = 38.23 ± 0.2 mm) were obtained from a hatchery in Central Finland. Fish were maintained in aerated ground water with continuous water flow (17°C) for 4 weeks before the experiments and fed daily with commercial fish food pellets. Prior to the exposures, the water temperature was raised slowly to 25°C (2°C every second day) to allow fish acclimation to experimental conditions. Three freshly grown strains of *F. columnare* (1–3, see Table S1) and clonally produced cercaria larvae of *D. pseudospathaceum* from three single‐genotype‐infected snails (A–C; see Table S2) were used in the fish exposures. Three hours prior to the exposures, the snails were placed individually in 2.5 dl of water (20°C) and allowed to produce cercariae. Cercarial density from each snail (fluke genotype) was estimated by counting ten times 1 ml subsamples from each container.

A pairwise infection design was then applied to test virulence and intensity of infection across the combinations (Table S3). In the experiment, 20 rainbow trout were exposed individually to single bacterial strains (5 × 10^3^ colony‐forming units/ml; 3 × 20 fish), single fluke genotypes (50 cercariae/fish; 3 × 20 fish) or co‐exposed to both bacteria and flukes in nine different combinations (9 × 20 fish), totalling 300 fish. To explore the effect of short‐term sequential infection on virulence, the co‐exposure matrix (9 × 20 fish) was replicated together with the simultaneous infections so that each of the nine co‐exposure combinations received the fluke first and the bacterium 4 hr later. Bacterial single infections (3 × 20 fish) were also repeated at this time with freshly grown strains to control for possible changes in bacterial virulence. A negative control group of 30 fish receiving pure culture medium and/or water instead of bacteria or flukes, respectively, was also established. Overall, the setup totalled 570 fish (Table S3). The infection doses corresponded to those in natural conditions. For example, fish infected with *F. columnare* can emit bacterial concentrations that are orders of magnitude higher than those used here (Kunttu, Valtonen, Jokinen, & Suomalainen, 2009) and one infected snail can release thousands of *D. pseudospathaceum* cercariae per day (Karvonen, Kirsi, Hudson, & Valtonen, 2004; Karvonen, Rellstab, Louhi, & Jokela, 2012). All fish were haphazardly assigned to the different treatment groups (single exposure to *F. columnare*, single exposure to *D. pseudospathaceum*, exposure to both parasites) in the simultaneous and sequential exposures.

The exposures and the subsequent monitoring took place in small containers with 500 ml preaerated ground water (25°C). The fish were checked every hour for disease symptoms and morbidity. Morbid fish that had lost their natural swimming buoyancy and did not respond to external stimuli were considered dead and were euthanized using MS‐222 anaesthetic every hour. This gave an accurate estimate of time of death (see Louhi et al., 2015). The fish were immediately sampled for presence of *F. columnare* on the skin and gills (by culture on Shieh containing tobramycin [Decostere et al., 1997]), and dissected for *D. pseudospathaceum* in the eye lenses. The establishment of *D. pseudospathaceum* in the eye lenses takes place within few hours from exposure (Louhi et al., 2015). The dissection protocol was used to determine the exact shape of the time‐establishment relationship used in estimation of differences in fluke abundance among the treatment groups (see below). The experiment was terminated at 28 hr post exposure when 87.5% of the fish exposed to the bacterium had died. All surviving fish were subsequently euthanized (MS‐222) and examined for bacterial and fluke infection as described above. Bacterial cultures confirming *F. columnare* infection were incubated at 22°C for 2 days and checked for presence of bacterial colonies.

Data on fish survival were analysed using Cox regression with sequential infection, *F. columnare* strain and *D. pseudospathaceum* genotype as fixed covariates, and fish length as a continuous covariate. Since the bacterial virulence changed slightly during the 4 hr interval between the infections (see results), fish groups exposed only to the bacterium at 0 hr and 4 hr were used as reference categories in the analysis. Thus, the effect of sequential infection on virulence would be seen as a significant three‐way interaction between the fixed factors. In addition, we applied analysis of covariance (ANCOVA) to data on fluke numbers in fish eyes to identify factors that affected infection intensity in exposures together with the bacterial strains. To correct for variation in fluke exposure and establishment time between fish individuals showing different time of survival, we used the residuals of the nonlinear asymptotic regression predicting infection intensity as function of survival time as the response variable (see Louhi et al., 2015).

### 2.4 Experimental exposure 2

To explore the longer‐term effect of sequential exposure, 960 rainbow trout from the same lot as in Experiment 1 were divided into 6 tanks, each with 160 fish and 70 L of water (16°C). Three of the groups were exposed to a total of 480 *D. pseudospathaceum* cercariae per tank, three cercariae per fish, while the other three groups served as unexposed controls. A low‐dose exposure was used to keep the number of parasites establishing in eye lenses low so that the parasite would not influence subsequent fish growth (Karvonen, 2012). Parasite cercariae were produced from two *L. stagnalis* snails as described above and pooled for the exposure (different genotypes to those used in the re‐exposure below (D–F), or in Experiment 1 [A–C]). During the exposure, the incoming water was turned off and was turned back on after 1 hr. As the cercarial infective lifespan is less than 30 hr (Karvonen, Paukku, Valtonen, & Hudson, 2003), parasites that failed to locate a fish, if any, were eventually lost from the tanks. Fish were then maintained for 5 weeks and fed daily with fish pellets. Possible acquired host responses against *D. pseudospathaceum* are cross‐reactive across parasite genotypes (Rellstab, Karvonen, Louhi, & Jokela, 2013), which minimized genotype‐specific responses, if any, between the first infection and the re‐exposure (see below). Water temperature was then raised slowly to 25°C as described above. Fish with and without the previous fluke infection were exposed either to single *Flavobacterium* (strains 1–3, Table S1; 2 × 3 × 20 fish), single *Diplostomum* (genotypes D–F, note different genotypes to Experiment 1 because of mortality among the snails carrying genotypes A–C, Table S2; 2 × 3 × 20 fish), or to pairwise combinations of the two (nine different combinations; 2 × 9 × 20 fish) (Table S4). Doses of the bacteria (5 × 10^3^ colony‐forming units/ml) and flukes (50 cercariae/fish) at re‐exposure were the same as in the first experiment. Each treatment had 20 replicate fish taken randomly from groups of previously unexposed and exposed fish (the three replicate tanks were pooled). A negative control group of 30 fish was also established. The entire setup totalled 630 fish (Table S4). Again, fish were maintained individually and followed for disease symptoms until 28 hr postexposure as described above. All fish that died or survived the experiment were sampled for bacterial presence of the skin and gills, and dissected for the number of flukes. Flukes originating from the first (three cercariae per fish) and the second (50 cercariae per fish) exposure were separated according to their size.

Data were analysed using Cox regression with initial fluke infection, *F. columnare* strain and *D. pseudospathaceum* genotype as fixed covariates, and fish length as a continuous covariate. Analysis of covariance (ANCOVA) was applied to residual fluke numbers as described above. All analyses were conducted in SPSS 24 statistical package. The experiments were approved by Finnish Regional State Administrative Agency (licence number ESAVI/1375/04.10.03/2012), and they conformed to the animal care legislation of Finland.

### 2.5 Modelling the population‐level effects of fluke infection on the impact of a bacterial epidemic

To explore the population‐level consequences of the individual‐level effects seen in Experiments 1 and 2, we developed a mathematical model similar to previously published models of priority effects in multiple infections (Clay, Cortez, Duffy, & Rudolf, 2019; Clay, Dhir, et al., 2019), parameterized with the data from our experiments, to predict the effects of prior or subsequent fluke infections on the impact of a bacterial epidemic within a single season (70 days) under aquaculture conditions. The model tracked changes in the proportion of hosts in the population that were either (a) infected with just the bacteria (“*B*”), (b) infected with just the fluke (“*F*”), (c) recovered from the bacteria infection (and assumed to be immune to bacterial reinfection during the same season; “*R*”), (d) sequentially infected with the fluke first, then the bacteria (“*C_FC_*”), (e) sequentially infected with the bacteria first, then the fluke (“*C_BC_*”), (f) previously infected with both parasites, but had recovered from their bacterial infection (but retained their fluke infection, and were immune to subsequent bacterial infection; “*F_R_*”), or (g) uninfected by either parasite (“*U*”).

Transitions between the various classes depended on the transmission and recovery rates of the bacteria and fluke. As stated above, hosts that recover from bacterial infection were assumed to be resistant against subsequent bacterial reinfection; bacteria‐only infected hosts (*B*) were assumed to recover to resistant hosts (*R*) at rate *σ_B_*, whereas sequentially infected hosts *C_FC_* and *C_BC_* recover to fluke‐infected resistant hosts (*F_R_*) at rates *σ_FC_* and *σ_BC_*, respectively. Hosts were assumed not to recover from fluke infections. Hosts susceptible to bacterial infections (i.e. all nonresistant hosts) were assumed to acquire bacterial infections at a rate dependent on the total abundance of all bacteria‐infected hosts (*B_T_ = B* + *C_FC_* + *C_BC_*), and *per capita* rate *β_B_*, resulting in the following transitions: *U* → *B* and *F* → *C_FC_*. Note, we assume these *per capita* transmission rates are the same regardless of fluke infection status, so prior or ongoing fluke infection is assumed not to influence bacterial infectivity or shedding rates. Because the fluke life cycle involves multiple life‐stages in different host species, spanning long durations, we modelled fluke transmission as a constant force of infection parameter (Γ; i.e. ignoring dynamic feedback between current infections and subsequent transmission rates), reflecting the population of cercariae present in the water throughout the season, resulting in the following transitions: *U* → *F*, *B* → *C_BC_* and *R* → *F_R_*. Hosts were assumed to die at infection‐specific mortality rates *μ_i_*, where *i* represents the infection class. Due to relative short duration of our simulations, we assumed no background mortality of uninfected fish or fluke‐only infected fish. Similarly, we assumed no increases in host population size (e.g. through host reproduction, immigration or input from external sources) corresponding to aquaculture conditions. Overall then, this leads to the following set of equations describing the changes in abundance of each host class:

$$\frac{\mathrm{dU}}{\mathrm{dt}}=-U\left(\beta_{B}B_{T}+\Gamma \right)$$

$$\frac{\mathrm{dB}}{\mathrm{dt}}=\beta_{B}B_{T}U-B\left(\mu_{B}+\Gamma +\sigma_{B}\right)$$

$$\frac{\mathrm{dF}}{\mathrm{dt}}=U\Gamma -F\beta_{B}B_{T}$$

$$\frac{\mathrm{dR}}{\mathrm{dt}}=B\sigma_{B}-R\Gamma$$

$$\frac{dC_{\text{FC}}}{\mathrm{dt}}=F\beta_{B}B_{T}-C_{\text{FC}}\left(\mu_{\text{FC}}+\sigma_{\text{FC}}\right)$$

$$\frac{dC_{\text{BC}}}{\mathrm{dt}}=B\Gamma -C_{\text{BC}}\left(\mu_{\text{BC}}+\sigma_{\text{BC}}\right)$$

$$\frac{dF_{R}}{\mathrm{dt}}=C_{\text{FC}}\sigma_{\text{FC}}+C_{\text{BC}}\sigma_{\text{BC}}+R\Gamma$$

We parameterized the model separately for each bacteria strain–fluke genotype combination using data from either Experiment 1 or Experiment 2, resulting in 18 parameter sets (3 bacterial strains × 3 fluke genotypes × 2 experiments). For each combination, the mortality rate of the appropriate infection classes (e.g. bacteria‐only infected hosts, sequential fluke–bacterial infection hosts) was given by the inverse of the observed mean host survival times for those experimental categories; sequential bacteria–fluke infection hosts were assumed to die at the rate given by the simultaneous infection experiments. The bacterial recovery rates for infection class *i* were calculated based on the observed proportion of fish surviving each experimental exposure (*p*_surv,_ *_i_*); assuming constant recovery and mortality rates of host class *i* are *σ_i_* and *μ_i_*, respectively, the expected proportion surviving is given by $p_{\text{surv},i}=\frac{\sigma_{i}}{\sigma_{i}+\mu_{i}}$. Since the *p*_surv,_ *_i_* are known for each infection class *i*, and the *μ_i_* can be estimated as described above, this equation can be rearranged to calculate the recovery rate $\sigma_{i}=\frac{p_{\text{surv},i}\cdot \mu_{i}}{\left(1-p_{\text{surv},i}\right)}$ that results in the observed proportion surviving from that class. The infection parameters in the system are unknown, so we chose an arbitrary value of bacterial transmission rate, *β_B_* (although we varied this value by two orders of magnitude around this baseline value and found no qualitative effect on our results), and varied the cercarial force of infection (Γ) to explore a range of scenarios of increasing fluke transmission pressure. All simulations were assumed to start with 100 fish, of which 10 were infected with the bacteria, to seed the epidemic. For each combination of bacterial strain × fluke genotype × experimental parameters, we ran the model for a duration of 70 days, and assessed the effect of varying cercarial infection pressure (Γ) on the end‐of‐season (day 70) total host abundance, compared to the scenarios when either (a) there was no bacterial epidemic, or (b) there was a bacterial epidemic, but no fluke infection. All models were run in R 3.5.1.

## 3 RESULTS

### 3.1 Experiment 1

**Figure 1:**
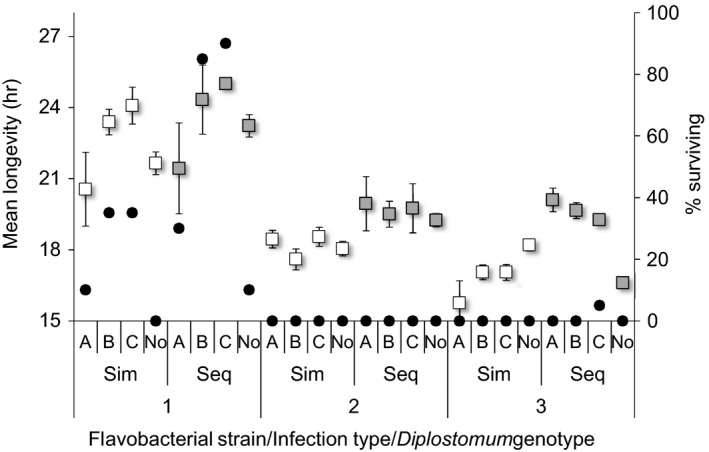
Mean survival times (±*SE*) of rainbow trout co‐exposed simultaneously (Sim, open boxes) or sequentially (Seq, grey boxes) to three strains of the bacterium *Flavobacterium columnare* (1–3) and three genotypes of the fluke *Diplostomum pseudospathaceum* (A–C) in all possible combinations in the first experiment. No *Diplostomum* indicates survival of fish exposed only to *F. columnare*. Boxes show data for fish that died during the experiment. Black dots indicate the percentage of fish surviving in each combination

Virulence of the flavobacterial strains differed significantly in single infections so that the strains 2 and 3 were more virulent compared to strain 1 [Cox regression: Wald = 70.48, *p* < .001 (strain)]. The virulence of the strains also slightly changed during the 4‐hr interval between the infections [Wald = 25.77, *p* < .001 (strain × sequence)] (Figure 1). *Diplostomum* infection alone did not cause mortality of fish. No mortality was observed either among the 30 unexposed control fish.

Sequential infection with the 4‐hr interval between the administrations of the two parasites significantly reduced the virulence of the secondary bacterial infection (Figure 1, Table 1). However, this was bacterial strain‐specific and most evident in strain 3 (interaction between sequential infection, flavobacterial strain and fluke genotype; Figure 1, Table 1). There was also a significant increase in the proportion of fish surviving the experiment with sequential infection in strain 1 when co‐exposed with fluke genotypes B [increase from 35% (95% CI = 15.4%–59.2%) to 85% surviving (62.1%–96.8%)] and C [35% (15.4%–59.2%) to 90% (68.3%–98.8%); Figure 1, Table S3].

**Table 1:** Results of stepwise Cox regression analyses on mortality of rainbow trout co‐exposed to three strains of the bacterium *Flavobacterium columnare* and three genotypes of the fluke *Diplostomum pseudospathaceum* in all possible combinations in Experiments 1 and 2

Experiment	Source	Wald	*df*	*p*	Exp (B)	95% CI
1	Sequential infection (S)	69.74	1	<.001	0.41	0.33–0.51
	G_B_	284.58	2	<.001		
	G_F_	51.32	3	<.001		
	S × G_F_	27.31	3	<.001		
	G_B_ × G_F_	35.26	6	<.001		
	S × G_B_ × G_F_	26.80	6	<.001		
2	Prior infection (I)	23.34	1	<.001	0.63	0.52–0.76
	G_B_	213.65	2	<.001		
	G_F_	8.94	3	.030		
	I × G_B_	7.56	2	.023		
	I × G_F_	14.25	3	.003		
	Fish length	10.55	1	.001		

Note

Infection type (simultaneous versus sequential (Exp 1) or no prior infection versus with prior infection [Exp 2]), bacterial strain (G_B_; 1–3) and fluke genotype (G_F_; A–C (Exp 1) or D–F [Exp 2]) were used as categorical covariates, and fish length as a continuous covariate.

The residual number of flukes was significantly different between the fluke genotypes, with genotype explaining large part of the variation (Figure S1, Table S5). Sequential infection did not affect the residual parasite numbers overall, but had an effect depending on the bacterial strain, fluke genotype and their interaction (Figure S1, Table S5). This suggests that the administration of the bacterium four hours later also affected the fluke numbers in strain–genotype‐specific manner.

### 3.2 Experiment 2

**Figure 2:**
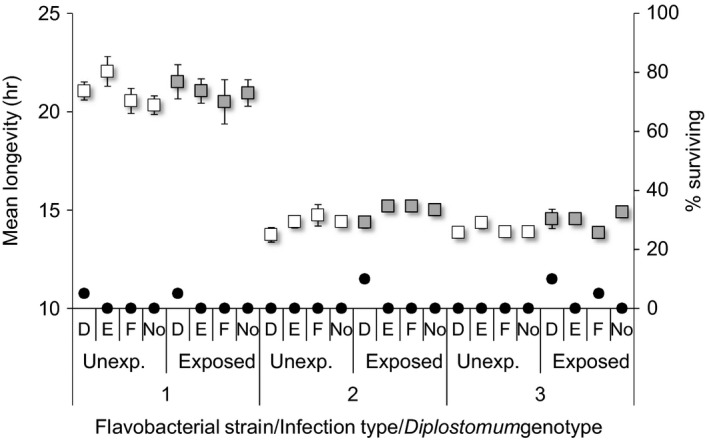
Mean survival times (±*SE*) of rainbow trout previously unexposed (Unexp., open boxes) or exposed to *Diplostomum pseudospathaceum* (Exposed, grey boxes) when re‐exposed to three strains of the bacterium *Flavobacterium columnare* (1–3) and three genotypes of the fluke *D. pseudospathaceum* (D–F) in all possible combinations in the second experiment. No *Diplostomum* indicates survival of fish exposed only to *F. columnare*. Boxes show data for fish that died during the experiment. Black dots indicate the percentage of fish surviving in each combination

Similarly to the Experiment 1, virulence of the flavobacterial strains 2 and 3 was higher compared to strain 1 [Cox regression: Wald = 39.61, *p* < .001 (strain)] and this pattern was independent of the previous exposure to flukes [Score = 1.33, *p* = .515 (prior infection × strain)] (Figure 2). Prior infection with flukes 5 weeks earlier resulted in an average of 1.68 ± 0.09 parasites established in the eyes of fish, which did not influence their growth compared to the uninfected fish (mean body length ± *SE*: 40.2 ± 0.2 mm (uninfected), 40.5 ± 0.2 mm (infected); *t* test: *t*_591_ = 1.069, *p* = .286). The infection caused a small, but significant reduction in the virulence of bacterial infection (Figure 2, Table 1). Again, the effect depended on the bacterial strain and was evident with strains 2 and 3 when co‐exposed with the fluke (Figure 2, Table 1). However, in single bacterial exposures, the effect of decreased virulence with the prior exposure to flukes was consistent across all strains (Wald = 5.472, *p* = .019; Figure 2). Single fluke infection did not cause significant mortality (two fish out of 120 exposed only to flukes died during the experiment). No mortality was observed among the 30 unexposed control fish. The residual number of flukes was significantly different between the fluke genotypes, indicating that genotypes differed in infection success. However, effects of the prior fluke infection, bacterial strain or their interactions on fluke numbers in the second exposure were not significant (Table S5).

### 3.3 Predicted population‐level effects of fluke infection on the impact of a bacterial epidemic

**Figure 3:**
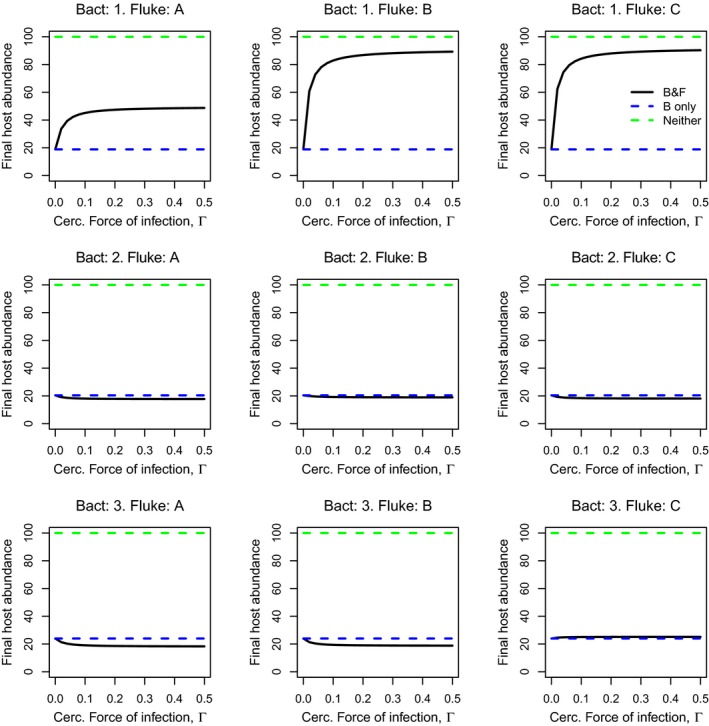
Model predictions of the end‐of‐season (day 70) total host abundance as a function of increasing cercarial force of infection (Γ), for each bacterial strain–fluke genotype combination, parameterized using data from Experiment 1. Solid black line = host abundance in the presence of both bacteria and fluke (“B&F”), dashed blue line = host abundance in the presence of just the bacteria (“B only”), dashed green line = host abundance in the absence of both bacteria and fluke (“Neither”). Other parameters: initial number of hosts = 100, initial number of bacteria‐infected hosts = 10, *β_B_* = 0.001

**Figure 4:**
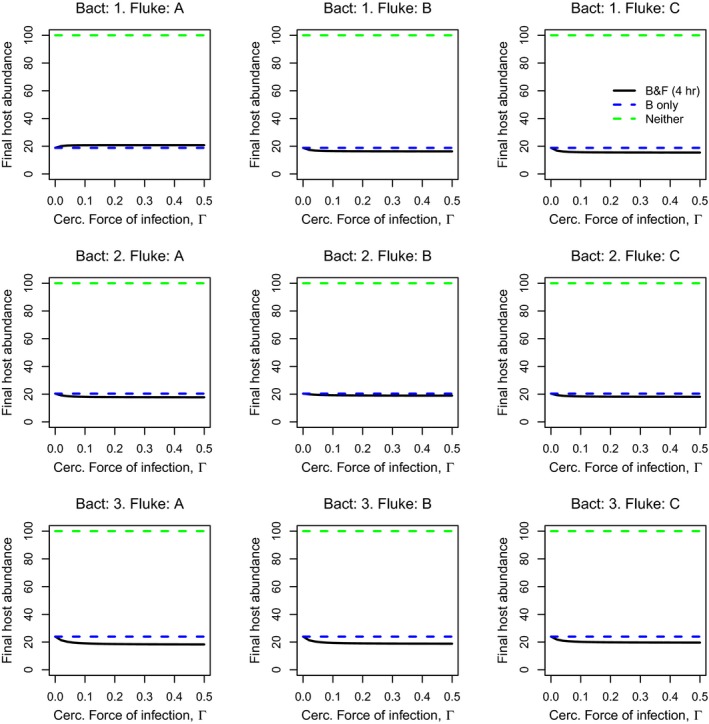
As in Figure 3, but ignoring any effect of fluke infection on recovery from bacterial infection (prior or subsequent fluke fish are assumed to recover from bacterial infection at the same rate as bacteria‐only infected fish; any effects of fluke infection on host survival time are retained, as in Figure 3)

Our model showed that the presence of fluke infections can protect the host population from the detrimental effect of a bacterial epidemic, most notably when using parameter values from Experiment 1, which assumed short‐term sequential infections (Figure 3). However, the magnitude of this protective effect varied considerably across bacterial strain and fluke genotype combinations; bacterial strain 1 (Figure 3, top row) appeared to be the most easily overcome strain, with increasing cercarial force of infection (Γ) leading to progressively higher levels of end‐of‐season fish abundance. The magnitude of these effects varied with fluke genotype, from around 40% at the highest levels of Γ examined for fluke genotype A, up to ~90% protection for fluke genotype C. However, population‐level protection from the other bacterial strains was negligible, regardless of the fluke genotype. Where population‐level protection was observed, (e.g. for bacterial strain 1), this was driven primarily by the increased recovery rate from bacterial infection of sequentially infected fish, as switching this component off (i.e. assuming recovery rates were the same regardless of the individual's prior fluke infection history) resulted in the loss of population‐level protection (Figure 4). Running the model using parameter values from Experiment 2, assuming longer‐term sequential infections, revealed low levels of population‐level protection, and now most commonly observed for fluke genotype D and for bacterial strain 3 (Figure S2).

## 4 DISCUSSION

Temporally variable infections of multiple parasites are common in the wild, potentially altering the outcomes of virulence in natural host–parasite interactions. Sequential parasite dynamics are common also in farming environments such as aquaculture, but the knowledge of the effects of temporal parasite dynamics on disease virulence in farmed animals is very limited. We investigated how temporal spacing between the infections of the pathogenic bacterium *F. columnare* and the fluke *D. pseudospathaceum* influenced the virulence of infection (morbidity) in aquaculture fish hosts. Both short (few hours) and long (several weeks) temporal differences between the infections resulted in reduction in bacterial virulence, while this effect depended on the genetic interactions among the parasite species. Similarly, timing of the infections changed the success of the fluke genotypes, suggesting influence also on parasite fitness. Overall, these results suggest that previous infections in different temporal scales can shape success and virulence of parasites with very different mechanisms of transmission and infection, and their subsequent impact on host population dynamics.

Previously, we have shown that the virulence of simultaneous infections of *F. columnare* and *D. pseudospathaceum* in fish is determined by complex genotype‐specific interactions (Louhi et al., 2015). Our present results, suggesting both short‐term and long‐term influences of sequential infections, add yet another dimension to these G × G interactions. Mechanistically, the lower virulence in sequential compared to simultaneous infections could be related, for example, to reduction in the rate of bacterial invasion to the host's body (Louhi et al., 2015), or to higher efficiency of the host's immune system to cope with two infections (Karvonen et al., 2012; Klemme et al., 2016). Similar host‐related factors could also influence the differences in fluke establishment, although detailed mechanisms underlying the changes in virulence and infection success are currently unclear. Overall, our results add significantly to earlier studies on sequential infections between different parasite taxa, majority of which have used single genotypes/strains (Ben‐Ami et al., 2011; Clay, Dhir, et al., 2019; Doublet et al., 2015; Hoverman et al., 2013; Lohr et al., 2010; Marchetto & Power, 2018; Natsopoulou et al., 2015), by emphasizing the importance of variation in infection outcomes depending on the specific G × G parasite combinations. Indeed, combining G × G interactions in multiple parasites with host infection history makes estimation of virulence and virulence evolution increasingly challenging (Karvonen, Jokela, & Laine, 2019). Nevertheless, such interactions could have important applied implications for scenarios of parasite prevention in intensive farming environments.

Our model on infection dynamics in an aquaculture fish population parameterized from the experimental data showed that a previous fluke infection, particularly few hours earlier, can protect the host population from the bacterial epidemic. However, this effect depended on the cercarial force of infection and, most importantly, on the bacteria–fluke genetic combinations. Interestingly, the protective effect increased dramatically at low cercarial forces of infection and was notably strong in some of the strain/genotype combinations, with up to ~90% of the population protected from the disease. This suggest that when flukes are present in the tank water, as a result of parasite input via incoming water or from infected snails inside the farm (Karvonen et al., 2006; Stables & Chappell, 1986), even a low‐level fluke infection could potentially decrease morbidity and mortality associated with an imminent bacterial exposure. This is consistent with earlier results of less‐virulent parasites providing host with at least some degree of protection against later arriving virulent strains/species in plants (Adame‐Alvarez, Mendiola‐Soto, & Heil, 2014; Seifi, Nonomura, Matsuda, Toyoda, & Bai, 2012; Tollenaere, Susi, & Laine, 2016) and invertebrates (Ben‐Ami, Mouton, et al., 2008; Clay, Cortez, et al., 2019; Wuerthner, Hua, & Hoverman, 2017). Similarly, the model on sequential infections 5 weeks apart predicted recovery in some of the parasite combinations, although the magnitude of this protective effect was clearly lower. While we could not compare the two experiments directly because they used different fluke genotypes, the experimental results and the model predictions suggest that the protective effect of sequential fluke infection against the bacterial outbreak might decay with time. If true, this could reflect operation of different short‐term and long‐term host‐level mechanisms such as reduction in parasite facilitation (Louhi et al., 2015) or activation of host immune system (Klemme et al., 2016) (see above). These are promising leads for future studies on the detailed mechanisms underlying the effects of sequential infections on disease epidemiology in this system.

Our model assumes single strain–genotype combinations between the parasites whereas in reality aquaculture fish would likely to be exposed to mixed genotypes of both flukes (Rauch, Kalbe, & Reusch, 2005) and bacteria (Kunttu et al., 2012; Sundberg et al., 2016), significantly increasing the number of possible genotype combinations and outcomes of the disease. Thus, the model predictions on effects of sequential infection in specific strain–genotype combinations can be considered as extremes, ranging from no effect to nearly total recovery of the host population. Given the likelihood of multiple strain–genotype infections, the reality is likely to lie somewhere in between these extremes, depending on the genetic composition of the bacterial and fluke populations in the water. Further, the model predictions were driven mainly by host recovery while our experimental data showed that sequential infection between the parasites also prolonged the lifetime of the hosts. While this effect was small in these experimental conditions, where the disease progression from exposure to morbidity is very fast (most fish had died within 28 hr), it could be speculated that the effect of increase in lifetime in aquaculture conditions, where the length of an untreated epidemic is typically several days or even weeks (Räihä, Sundberg, Ashrafi, Hyvärinen, & Karvonen, 2019), could be stronger. Importantly, these increases in infected host lifetime, though beneficial to the individual host, could have counter‐productive effects on the host population as a whole. Similar to host “tolerance” responses to infection (Ayres & Schneider, 2012; Medzhitov, Schneider, & Soares, 2012; Råberg, Graham, & Read, 2009), by keeping infected hosts alive, this prolongs the window of opportunity for infection to other hosts in the population, increasing overall infection prevalence, and resulting in a net decrease in host population abundance (e.g. Vale, Fenton, & Brown, 2014), and, as most clearly seen here, in the absence of any effect of fluke infection on host recovery from bacterial infection (Figure 4). However, the detailed epidemiological consequences of the prolonged lifetime are currently unknown and require further work. Finally, competition/interactions between the bacterial strains (Kinnula, Mappes, & Sundberg, 2017; Sundberg et al., 2016), in interaction with the flukes infecting the hosts simultaneously (Louhi et al., 2015) or sequentially (this study), could also shape the evolution of virulence. For example, as the order of parasite arrival to host can significantly alter the outcome of virulence (Alizon, de Roode, & Michalakis, 2013), factors such as G × G interactions between parasites could be important in determining which virulence genotypes are favoured by selection under each infection scenario (Karvonen, Jokela, et al., 2019). However, while our results support G × G variation in infection outcomes, a formal study of implications of sequential infections on evolution of virulence is beyond the scope of the present model/study.

To conclude, flavobacteria are currently considered among the most important bacterial fish pathogens worldwide. Interactions between increasingly virulent strains of *F. columnare* (Kinnula et al., 2017; Pulkkinen et al., 2010; Sundberg et al., 2016) and those between the bacterium and other co‐occurring parasite species (Bandilla, Valtonen, Suomalainen, Aphalo, & Hakalahti, 2006; Louhi et al., 2015; Xu, Shoemaker, & LaFrentz, 2014) can significantly alter the disease outcomes and influence predictions on virulence of infection. Our results, suggesting protective effect of sequential infections from a less‐virulent parasite against a highly virulent one (see also King et al., 2016), provide an interesting viewpoint into how epidemics could be altered by the host infection history. While the epidemiological effects of consecutive parasite outbreaks on subsequent disease occurrence are generally poorly known, results from our model suggest that changes in disease epidemiology with sequential exposure can also have important implications for the need and success of disease management practices and medication protocols. In a wider perspective, possible reduction in use of medication could also constrain environmental discharge of medical residues and pathogen evolution for antibiotic resistance (Cabello, 2006; Martinez, 2009).

## CONFLICT OF INTEREST

We have no competing interests.

## Data Availability

Data are archived in Dryad Digital Repository. https://doi.org/10.5061/dryad.8f57ck3 (Karvonen, Fenton, & Sundberg, 2019).
