# Supplementary material for: Sequential infection can decrease virulence in a fish–bacterium–fluke interaction: Implications for aquaculture disease management
Source: Evol Appl. 2019 Aug 14;12(10):1900–11. doi: 10.1111/eva.12850 (PMC6824072; doi:10.1111/eva.12850)

Karvonen, A., Fenton, A., Sundberg, L.-R. Sequential infection can decrease virulence in a fish-bacterium-fluke interaction: implications for aquaculture disease management

Supplementary material

Table S1. *Flavobacterium columnare* strains used in the study. Strains were originally isolated from tank water of a fish farm or from river water, in 2008-2010. ARISA group refers to the Automated Ribosomal Intergenic Spacer analysis used in genetic characterization of bacterial strains.

Strain	Original code	Isolation source and year	ARISA group	Reference
1	B185	Fish farm, tank water, 2008	G	[1]
2	B245	Fish farm, tank water, 2009	C	[1]
3	B406	River water, 2010	C	[2]

References:

[1] Laanto E., Bamford J.K., Laakso J. & Sundberg L.-R. 2012 Phage-driven loss of virulence in a fish pathogenic bacterium. *PLoS ONE*, *7*, e53157.
[2] Kunttu H.M., Sundberg L.-R., Pulkkinen K. & Valtonen E.T. 2012 Environment may be the source of *Flavobacterium columnare* outbreaks at fish farms. *Environmental Microbiology Reports*, *4*, 398-402.

Table S2. Result of the microsatellite analyses on 15 individual cercariae of *Diplostomum pseudospathaceum* from six snails infected with one parasite genotype. Numbers refer to different alleles at each loci. Details of the genotyping process are described in Louhi et al. (2010).

Snail ID	N cercariae successfully genotyped	Loci Diplo06	Loci Diplo09	Loci Diplo23
A	14/15	90/139	174/191	110/123
B	15/15	88/137	176/214	110/116
C	14/15	91/115	184/207	106/119
D	15/15	90/102	176/193	114/116
E	15/15	96/100	170/174	110/127
F	15/15	92/105	213/216	106/108

Reference:

Louhi, K.-R., Karvonen, A., Rellstab, C., & Jokela, J. (2010). Is the population genetic structure of complex life cycle parasites determined by the geographic range of the most motile host? *Infection, Genetics and Evolution, 10*, 1271-1277. doi:10.1016/j.meegid.2010.08.013

Table S3. Design of the Experiment 1. Young rainbow trout were exposed only to *Flavobacterium columnare*, only to *Diplostomum pseudospathaceum* flukes, or to both parasites, either simultaneously or sequentially with the fluke presented first and the bacterium 4 h later. Three different *F. columnare* strains (1-3) and three different genotypes of *D. pseudospathaceum* (A-C) were used. Columns show the mean survival time of the fish that died (standard error) and the proportion of fish surviving (95% confidence interval) for all exposure combinations and for the unexposed controls.

Flavobacterium strain	Type of infection	Diplostomum genotype	N fish	Mean survival time (h)	SE	Proportion fish surviving	95% CI
1	Simultaneous	A	20	20.56	1.56	0.1	0.012-0.317
		B	20	23.38	0.54	0.35	0.154-0.592
		C	20	24.08	0.78	0.35	0.154-0.592
		No Diplostomum, bacterium only	20	21.65	0.48	0	0-0.168
	Sequential	A	20	21.43	1.91	0.3	0.119-0.543
		B	20	24.33	1.45	0.85	0.621-0.968
		C	20	25.00	0.00	0.9	0.683-0.988
		No Diplostomum, bacterium only	20	23.22	0.48	0.1	0.012-0.317
2	Simultaneous	A	20	18.45	0.37	0	0-0.168
		B	20	17.60	0.44	0	0-0.168
		C	20	18.55	0.40	0	0-0.168
		No Diplostomum, bacterium only	20	18.05	0.30	0	0-0.168
	Sequential	A	20	19.95	1.15	0	0-0.168
		B	20	19.50	0.55	0	0-0.168
		C	20	19.75	1.03	0	0-0.168
		No Diplostomum, bacterium only	20	19.25	0.28	0	0-0.168
3	Simultaneous	A	20	15.75	0.95	0	0-0.168
		B	20	17.05	0.31	0	0-0.168
		C	20	17.05	0.34	0	0-0.168
		No Diplostomum, bacterium only	20	18.20	0.25	0	0-0.168
	Sequential	A	20	20.10	0.50	0	0-0.168
		B	20	19.65	0.33	0	0-0.168
		C	20	19.26	0.23	0.05	0.001-0.249
		No Diplostomum, bacterium only	20	16.60	0.18	0	0-0.168
No Flavobacterium	Diplostomum only	A	20	-	-	1	0.832-1
		B	20	-	-	1	0.832-1
		C	20	-	-	1	0.832-1
No Flavobacterium	Unexposed control	No Diplostomum	30	-	-	1	0.884-1

Table S4. Design of the Experiment 2. Young rainbow trout, with and without a previous infection with the fluke *D. pseudospathaceum*, were exposed only to *F. columnare*, only to *D. pseudospathaceum*, or simultaneously to both parasites. Three different *F. columnare* strains (1-3) and three different genotypes of *D. pseudospathaceum* (D-F) were used. Columns show the mean survival time of the fish that died (standard error) and the proportion of fish surviving (95% confidence interval) for all exposure combinations and for the unexposed controls.

Flavobacterium strain	Previous exposure to flukes	Diplostomum genotype	N fish	Mean survival time (h)	SE	Proportion fish surviving	95% CI
1	No	D	20	21.05	0.46	0.05	0.001-0.249
		E	20	22.05	0.76	0	0-0.168
		F	20	20.55	0.63	0	0-0.168
		No Diplostomum, bacterium only	20	20.33	0.47	0	0-0.168
	Yes	D	20	21.53	0.87	0.05	0.001-0.249
		E	20	21.05	0.62	0	0-0.168
		F	20	20.50	1.13	0	0-0.168
		No Diplostomum, bacterium only	20	20.95	0.67	0	0-0.168
2	No	D	20	13.75	0.37	0	0-0.168
		E	20	14.40	0.30	0	0-0.168
		F	20	14.74	0.55	0	0-0.168
		No Diplostomum, bacterium only	20	14.40	0.18	0	0-0.168
	Yes	D	20	14.39	0.30	0.1	0.012-0.317
		E	20	15.20	0.22	0	0-0.168
		F	20	15.20	0.24	0	0-0.168
		No Diplostomum, bacterium only	20	15.00	0.27	0	0-0.168
3	No	D	20	13.85	0.26	0	0-0.168
		E	20	14.35	0.29	0	0-0.168
		F	20	13.90	0.20	0	0-0.168
		No Diplostomum, bacterium only	20	13.90	0.26	0	0-0.168
	Yes	D	20	14.56	0.49	0.1	0.012-0.317
		E	20	14.55	0.22	0	0-0.168
		F	20	13.84	0.22	0.05	0.001-0.249
		No Diplostomum, bacterium only	20	14.90	0.23	0	0-0.168
No Flavobacterium	No	D	20	-	-	1	0.832-1
		E	20	-	-	1	0.832-1
		F	20	-	-	1	0.832-1
	Yes	D	20	7.00	0.00	0.95	0.751-0.999
		E	20	10.00	0.00	0.95	0.751-0.999
		F	20	-	-	1	0.832-1
No Flavobacterium	Unexposed control	No Diplostomum	30	-	-	1	0.884-1

Table S5. Results of ANCOVA on residual numbers of flukes in fish co-exposed to three strains of the bacterium *Flavobacterium columnare* and three genotypes of the fluke *Diplostomum pseudospathaceum* in all possible combinations in the experiments 1 and 2. Infection type [simultaneous vs. sequential (Exp 1), or no prior fluke infection vs. with prior fluke infection (Exp 2)], bacterial strain (G_B; 1-3) and fluke genotype [G_F; A-C (Exp 1) or D-F (Exp 2)] were used as fixed factors, and fish length as a covariate.

**Experiment**	**Source**	**MS**	**d.f.**	**F**	**p**	**η^2**
1	Sequential infection (S)	31	1	0.56	0.455	0.002
	G_B	454	2	8.22	<0.001	0.046
	G_F	6107	2	110.65	<0.001	0.395
	$S \times G_B$	379	2	6.86	0.001	0.039
	$S \times G_F$	258	2	4.68	0.010	0.027
	$G_B \times G_F$	177	4	3.21	0.013	0.036
	$S \times G_B \times G_F$	42	4	0.75	0.557	0.009
	Length	827	1	14.97	<0.001	0.042
	Error	55	339			
2	Prior infection (I)	196	1	2.626	0.106	0.008
	G_B	155	2	2.083	0.126	0.012
	G_F	1841	2	24.657	<0.001	0.127
	$I \times G_B$	67	2	0.903	0.406	0.005
	$I \times G_F$	48	2	0.637	0.529	0.004
	$G_B \times G_F$	24	4	0.321	0.864	0.004
	$I \times G_B \times G_F$	51	4	0.685	0.603	0.008
	Length	285	1	3.817	0.052	0.011
	Error	75	338			

Fig. S1. Estimated mean residual number of flukes *Diplostomum pseudospathaceum* (±SE) in rainbow trout co-exposed simultaneously (Sim, open boxes) or sequentially (Seq, grey boxes) to three genotypes of the fluke (A-C) and three strains of the bacterium *Flavobacterium columnare* (1-3) in all possible combinations in the first experiment. Residuals are from a non-linear asymptotic regression predicting infection intensity as function of survival time and estimations are from an ANCOVA model (Table S5).

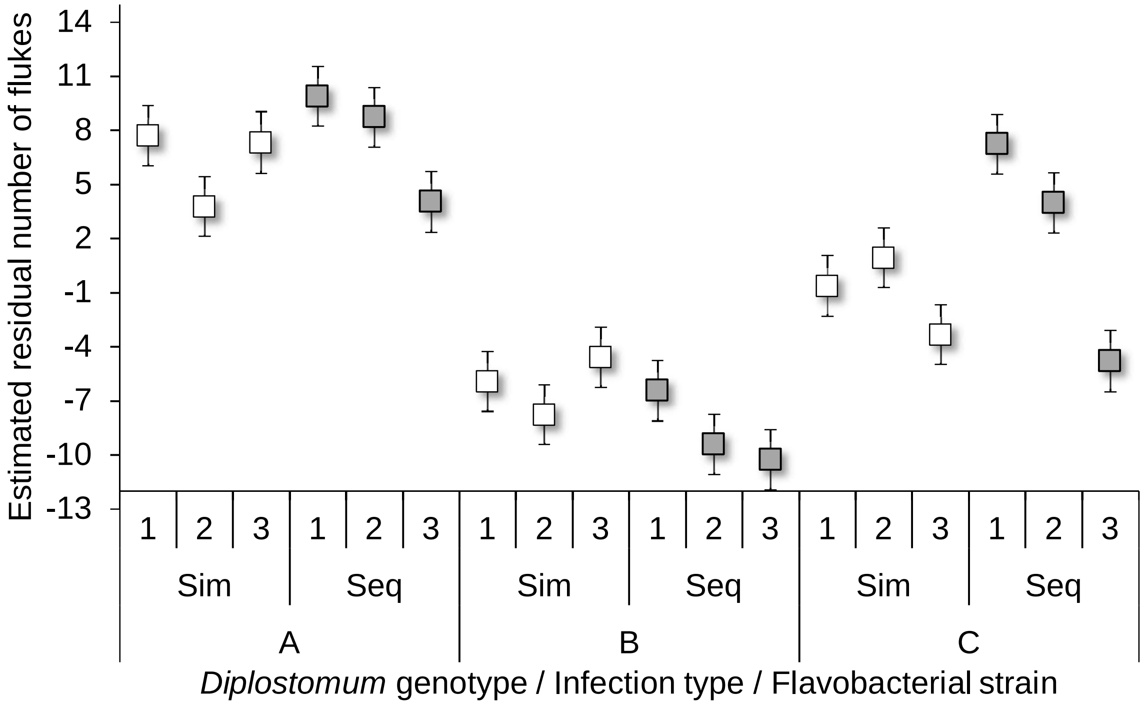

Fig. S2. As in Fig. 3, but parameterized using survival and recovery rates from Experiment 2, assuming long-term effects of sequential infection.

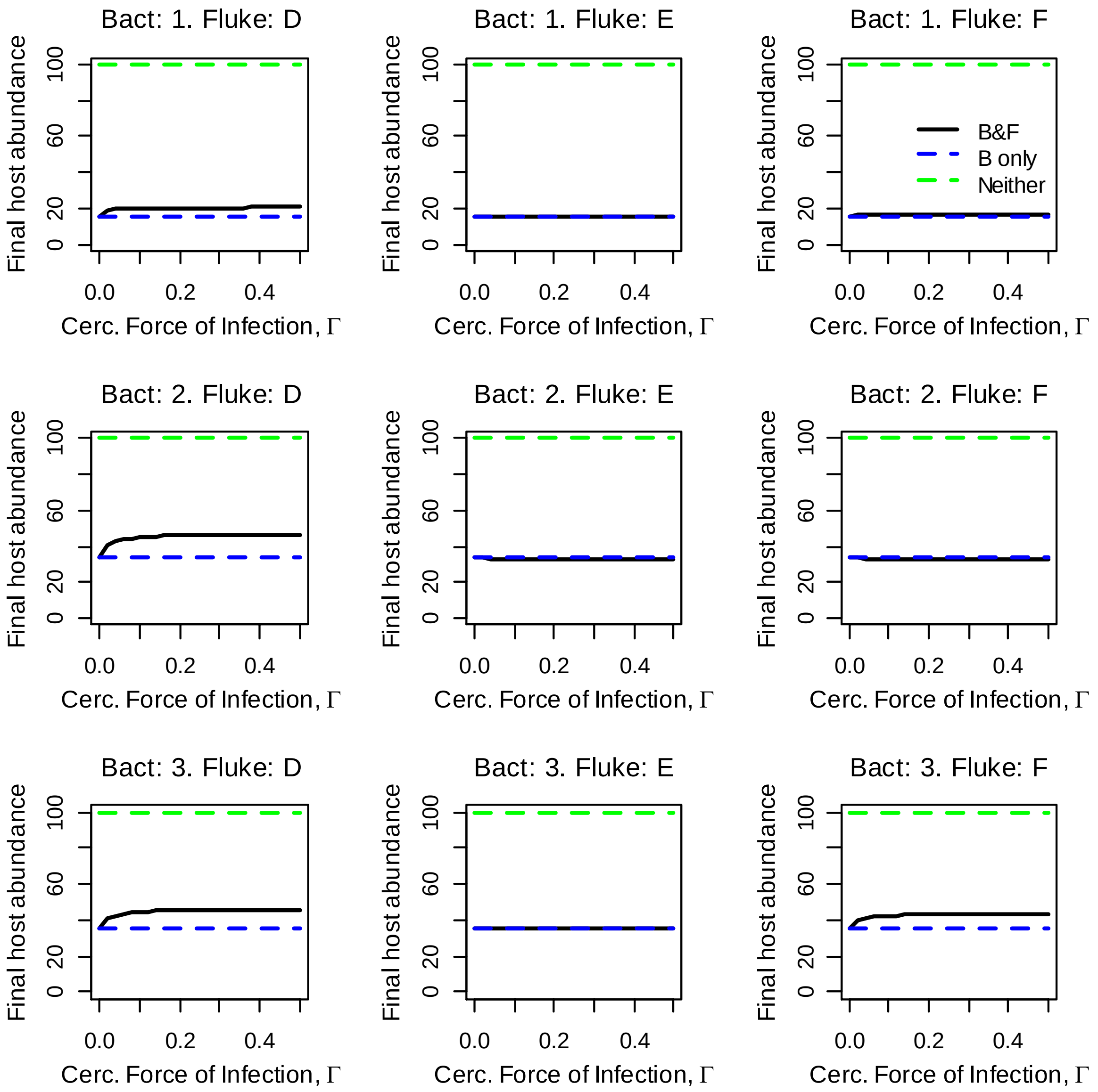

Supplement: Supplementary file 1 [file EVA-12-1900-s001.pdf]
